# In this month’s Bulletin

**DOI:** 10.2471/BLT.19.000919

**Published:** 2019-09-01

In the editorial section, Amir Aman Hagos et al. (582) introduce a recent World Health Assembly resolution on emergency care systems.

In the news section, Lynn Eaton (585–586) reports on burn-out as an occupational health issue for health care workers. Teguest Guerma talks to Gary Humphreys (587–588) about the early years of the HIV epidemic and her current commitment to training midwives in Ethiopia. 

## Bangladesh

### Counting everyone

Moyeen Uddin et al. (637–641) describe a pilot programme to increase civil registration coverage. 

## Benin, Mali, Mozambique and Togo

### How to pay for universal health coverage

Inke Mathauer et al. (620–630) explore options for raising revenue. 

## Indonesia

### Managing acute malnutrition 

Blandina Rosalina Bait et al. (597–604) report implementation research from Kupang district.

## Japan

### Labour and delivery

Yuto Maeda et al. (631–636) explain shortfalls in analgesia during childbirth.

## Thailand

### Meeting national needs

Sirinad Tiantong et al. (642–644) create a new cooperation strategy.

## Global

### Deaths before and after 70

Peter Byass (589–596) examines noncommunicable disease mortality correlates.

### Harm reduction for people who inject drugs

Daniel O’Keefe et al. (605–611) argue for better indicators of access and impact.

### Emergency care systems

Taylor W Burkholder et al. (612–619) propose a rights-based approach. 

